# Acute abdomen in the immunocompromised patient: WSES, SIS-E, WSIS, AAST, and GAIS guidelines

**DOI:** 10.1186/s13017-021-00380-1

**Published:** 2021-08-09

**Authors:** Federico Coccolini, Mario Improta, Massimo Sartelli, Kemal Rasa, Robert Sawyer, Raul Coimbra, Massimo Chiarugi, Andrey Litvin, Timothy Hardcastle, Francesco Forfori, Jean-Louis Vincent, Andreas Hecker, Richard Ten Broek, Luigi Bonavina, Mircea Chirica, Ugo Boggi, Emmanuil Pikoulis, Salomone Di Saverio, Philippe Montravers, Goran Augustin, Dario Tartaglia, Enrico Cicuttin, Camilla Cremonini, Bruno Viaggi, Belinda De Simone, Manu Malbrain, Vishal G. Shelat, Paola Fugazzola, Luca Ansaloni, Arda Isik, Ines Rubio, Itani Kamal, Francesco Corradi, Antonio Tarasconi, Stefano Gitto, Mauro Podda, Anastasia Pikoulis, Ari Leppaniemi, Marco Ceresoli, Oreste Romeo, Ernest E. Moore, Zaza Demetrashvili, Walter L. Biffl, Imitiaz Wani, Matti Tolonen, Therese Duane, Sameer Dhingra, Nicola DeAngelis, Edward Tan, Fikri Abu-Zidan, Carlos Ordonez, Yunfeng Cui, Francesco Labricciosa, Gennaro Perrone, Francesco Di Marzo, Andrew Peitzman, Boris Sakakushev, Michael Sugrue, Marja Boermeester, Ramiro Manzano Nunez, Carlos Augusto Gomes, Miklosh Bala, Yoram Kluger, Fausto Catena

**Affiliations:** 1 General, Emergency and Trauma Surgery Department, Pisa University Hospital, Via Paradisa, 2, 56124 Pisa, Italy; 2 Emergency Department, Pavia University Hospital, Pavia, Italy; 3 General and Emergency Macerata Hospital, Macerata, Italy; 4 Department of Surgery, Anadolu Medical Center, Kocaali, Turkey; 5 General Surgery Department, Western Michigan University, Kalamazoo, MI USA; 6 Department of General Surgery, Riverside University Health System Medical Center, Moreno Valley, CA USA; 7 Department of Surgical Disciplines, Immanuel Kant Baltic Federal University, Kaliningrad, Russia; 8 Emergency and Trauma Surgery, Inkosi Albert Luthuli Central Hospital, Mayville, South Africa; 9 Intensive Care Unit, Pisa University Hospital, Pisa, Italy; 10 Departement of Intensive Care, Erasme Univ Hospital, Université Libre de Bruxelles, Bruxelles, Belgium; 11 Departementof General and Thoracic Surgery, University Hospital of Giessen, Giessen, Germany; 12 General Surgery, Radboud University Medical Center, Nijmegen, The Netherlands; 13 General Surgery, San Donato Hospital, Milano, Italy; 14 General Surgery, CHUGA-CHU Grenoble Alpes UGA-Université Grenoble Alpes, Grenoble, France; 15 General Surgery, Pisa University Hospital, Pisa, Italy; 16 3rd Department of Surgery, Attiko Hospital, National & Kapodistrian University of Athens, Athens, Greece; 17 General Surgery, Varese University Hospital, Varese, Italy; 18 Département d’Anesthésie-Réanimation, CHU Bichat Claude Bernard, Paris, France; 19 Department of Surgery, Zagreb University Hospital Centre and School of Medicine, University of Zagreb, Zagreb, Croatia; 20 ICU Department, Careggi University Hospital, Firenze, Italy; 21 Department of Digestive, Metabolic and Emergency Minimally Invasive Surgery, Centre Hospitalier Intercommunal de Poissy/Saint Germain en Laye, Saint Germain en Laye, France; 22 Faculty of Engineering, Department of Electronics and Informatics, Vrije Universiteit Brussel, Brussels, Belgium; 23 General and Emergency Surgery, Tan Tock Seng Hospital, Kuala Lumpur, Malaysia; 24 General and Emergency Surgery, Pavia University Hospital, Pavia, Italy; 25 General Surgery, School of Medicine, Istanbul Medeniyet University, Istanbul, Turkey; 26 Department of General Surgery, La Paz University Hospital, Madrid, Spain; 27 General Surgery, VA Boston Health Care System, Boston University, Harvard Medical School, Boston, MA USA; 28 General Surgery, Parma University Hospital, Parma, Italy; 29 Gastroenterology and Transplant Unit, Firenze University Hospital, Firenze, Italy; 30 General and Emergency Surgery, Cagliari University Hospital, Cagliari, Italy; 31 Medical Department, National & Kapodistrian University of Athens, Athens, Greece; 32 Abdominal Center, Helsinki University and Helsinki University Hospital, Helsinki, Finland; 33 General Surgery, Monza University Hospital, Monza, Italy; 34 Department of Surgery, Western Michigan University School of Medicine, Kalamazoo, MI USA; 35 Trauma Surgery, Denver Health, Denver, CL USA; 36 General Surgery, Tbilisi State Medical University, Tbilisi, Georgia; 37 Emergency and Trauma Surgery, Scripps Memorial Hospital La Jolla, La Jolla, CA USA; 38 General Surgery, Government Gousia Hospital, Srinagar, Kashmir India; 39 Envision Healthcare, Dallas, TX USA; 40 National Institute of Pharmaceutical Education and Research, Hajipur (NIPER-H), Vaishali, Bihar India; 41 General Surgery Department, Henry Mondor University Hospital, Paris, France; 42 Emergency Medicine, Radboud University Medical Center, Nijmegen, The Netherlands; 43 General Surgery, UAE University Hospital, Sharjah, United Arab Emirates; 44 Division of Trauma and Acute Care Surgery, Department of Surgery, Fundación Valle del Lili, Universidad del Valle, Cali, Colombia; 45 Department of Surgery, Tianjin Nankai Hospital, Nankai Clinical School of Medicine, Tianjin Medical University, Tianjin, China; 46 Global Alliance for Infections in Surgery, Vila Nova de Gaia, Portugal; 47 General Surgery, Valtiberina Hospital, Sansepolcro, Italy; 48 General Surgery, University of Pittsburgh School of Medicine, Pittsburgh, USA; 49 First Clinic of General Surgery, University Hospital St George Plovdiv, Plovdiv, Bulgaria; 50 General Surgery, Letterkenny Hospital, Letterkenny, Ireland; 51 Department of Surgery, Academic Medical Center, Amsterdam, Netherlands; 52 Clinical Research Center, Fundacion Valle del Lili, Cali, Colombia; 53 Department of Surgery, Faculdade de Ciências Médicas e da Saúde de Juiz de Fora, Hospital Universitário Terezinha de Jesus, Juiz de Fora, Brazil; 54 General Surgery, Hadassah Hospital, Jerusalem, Israel; 55 General Sugery, Ramabam Medical Centre, Tel Aviv, Israel

**Keywords:** Infections, Intra-abdominal, Peritonitis, Cholecystitis, Appendicitis, Diverticulitis, Perforation, Transplanted, Oncologic, Cancer, Perioperative, Anesthesia, Hematologic, Cytomegalovirus, Tuberculosis, Lymphoma, Leukemia, Immunosuppression, Immunocompetence, Immunocompromise

## Abstract

Immunocompromised patients are a heterogeneous and diffuse category frequently presenting to the emergency department with acute surgical diseases. Diagnosis and treatment in immunocompromised patients are often complex and must be multidisciplinary. Misdiagnosis of an acute surgical disease may be followed by increased morbidity and mortality. Delayed diagnosis and treatment of surgical disease occur; these patients may seek medical assistance late because their symptoms are often ambiguous. Also, they develop unique surgical problems that do not affect the general population. Management of this population must be multidisciplinary.

This paper presents the World Society of Emergency Surgery (WSES), Surgical Infection Society Europe (SIS-E), World Surgical Infection Society (WSIS), American Association for the Surgery of Trauma (AAST), and Global Alliance for Infection in Surgery (GAIS) joined guidelines about the management of acute abdomen in immunocompromised patients.

## Introduction

Emergency surgery admissions carry a substantial risk of in-hospital death of 3.04% [1] and a chance of postoperative complication of 21%. That is further increased with an immunocompromised state. Immunocompromised patients (IP) are a heterogeneous and diffuse category of patients frequently presenting to the emergency department (ED) with acute surgical diseases. Diagnosis and treatment in IP are often challenging and must be multidisciplinary. Misdiagnosing of acute surgical disease in an IP may be followed by increased morbidity and mortality. IP not only seek later medical assistance because their symptoms are often undefined, but they have some unique surgical problems that do not affect the general population.

There have been a few attempts to stratify these patients in the last 30 years, especially since a universally accepted definition of an immunocompromised state does not exist [2, 3].

Revision of all those conditions and diseases causing immunocompromission (IC) may lead to patient categorization into two groups: one with mild-moderate IC and another with severe IC (Table 1). Precise indications deriving from the literature are scarce. The present paper represents the World Society of Emergency Surgery (WSES), Surgical Infection Society Europe (SIS-E), World Surgical Infection Society (WSIS), American Association for the Surgery of Trauma (AAST), and Global Alliance for Infection in Surgery (GAIS) joined guidelines about the management of acute abdomen in immunocompromised patients.

**Table 1:** Clinical classification of patients with immune deficiency

**Mild-moderate immune deficiency**	
Elderly (according to the age and general status of the patient)	
Malnourished	
Diabetic	
Burns	
Trauma	
Uremic	
Active malignancy, not on chemotherapy	
HIV with CD4+ count > 200/mm^3^	
Splenectomized	
**Severe immune deficiency**	
AIDS	
HIV with CD4+ count < 200/mm^3^	
Transplant (solid organ, bone marrow)	
High-dose steroids (more than 20 mg/day prednisone)	
Malignancy on chemotherapy	
Neutrophil count < 1000/mm^3^	

## Material and methods

### Research strategy

**Fig. 1:**
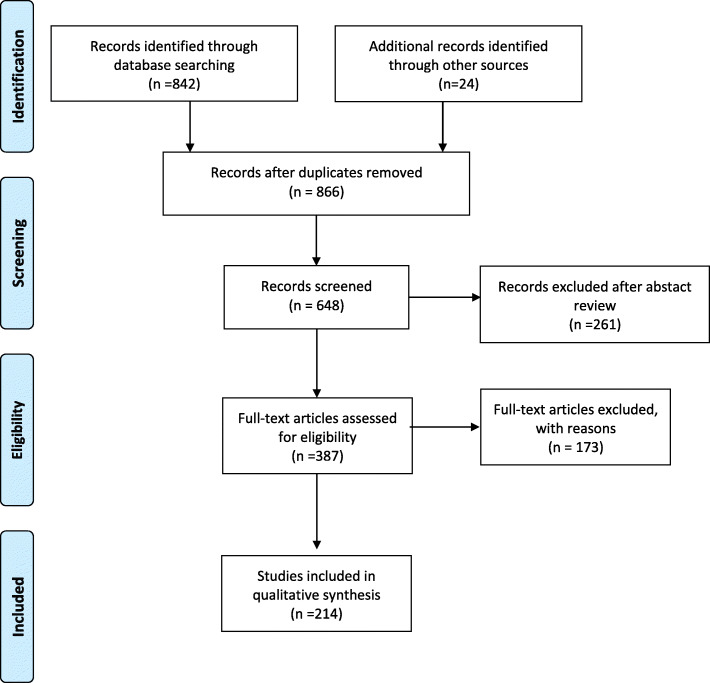
Prisma flowchart

The bibliographer conducted a computerized search in different databanks (MEDLINE, PubMed, Scopus, Web of Science, EMBASE). Citations were included for the period between January 1990 and March 2020 using the primary search strategy: emergency surgery, general, immunocompromised, immunosuppressed, abdominal sepsis, infection, with AND/OR. As the definition of immunocompromission is quite variable, the search also included terms as “HIV”, “AIDS”, “transplanted”, and “chronic steroid therapy” with synonyms and MeSH terms. No language restriction was imposed. Duplicates and animal studies were removed. The dates were selected to allow comprehensive published abstracts of clinical trials, consensus conferences, comparative studies, congresses, guidelines, government publication, multicenter studies, systematic reviews, meta-analysis, large case series, original articles, and randomized controlled trials. Narrative review articles were also analyzed to identify other studies. Abstracts were screened, and not relevant studies were removed; then, a full-text assessment of the articles was performed. Case reports were excluded. In case of disagreement between the two reviewers (FC, MI), the consensus was reached by discussion. If there was no consensus, a third reviewer was sought (FCa). Prisma flowchart of the systematic review is reported in Fig. 1.

Level of evidence (LoE) graded in high, moderate, low, and very low and the grade of recommendation (GoR) graded as strong, moderate, and weak were calculated according to the WSES rules for guidelines update, keeping into consideration the GRADE model [4].

An international expert panel in a modified Delphi process discussed the different issues in subsequent rounds. At each round, the manuscript was revised and improved. The final version about which agreement was reached resulted in the present manuscript. Statements are summarized in Table 2.

**Table 2:** Statements’ summarizing table

	Statements
**Diagnosis**	- Diagnosis and treatment in immunocompromised patients must be multidisciplinary (GoR moderate based on low LoE). - High clinical suspicion must be kept in the presence of an immunocompromised patient presenting with signs and/or symptoms of possible intrabdominal infection (GoR moderate based on low LoE). - Immunocompromised patients usually do not present specific signs and symptoms. A reliable diagnosis may be reached only by combining signs, symptoms, patient history, and radiological evaluation (GoR moderate based on low LoE). - Clinical signs may not be reliable in immunocompromised patients; the more the immunocompromission, the less the reliability (GoR moderate based on low LoE). - Laboratory tests may not accurately reflect the severity of the clinical condition of the patient immunocompromised (GoR moderate based on low LoE). - Plane radiographs and ultrasound are often not sufficiently sensitive and specific to allow for a definitive diagnosis in immunocompromised patients (GoR moderate based on low LoE). - Contrast-enhanced CT scan, whenever feasible, is the most reliable exam to diagnose intrabdominal disease in immunocompromised patients (GoR moderate based on low LoE). - In the event of diarrhea, with or without acute abdomen, a specific test for *Clostridioides difficile* and its toxin should be performed (GoR moderate based on low LoE). - Additional microbiologic tests for a specific disease should be performed only if clinically congruent (GoR moderate based on low LoE). - Diagnostic workup for acute abdomen in patients with HIV infection should always consider surgical diseases specifically associated with HIV (i.e., Abdominal tuberculosis, Mycobacterium avium complex infections) (GoR moderate based on low LoE).
**Specific acute abdominal infections in immunocompromised patient**	
**Neutropenic enterocolitis**	- Neutropenic enteritis and typhlitis have a high mortality rate if misdiagnosed or underestimated; accurate differential diagnosis is mandatory (GoR moderate based on low LoE). - Treatment of neutropenic enteritis and typhlitis should be nonoperative, including broad-spectrum antibiotics and bowel rest. Emergency surgery must be reserved only for those patients presenting with signs of perforation or ischemia (GoR moderate based on low LoE). - A damage control approach in complicated neutropenic enteritis and typhlitis should be adopted in severely sick patients with physiological derangement (GoR moderate based on low LoE).
**Cytomegalovirus colitis**	- Cytomegalovirus colitis has a high mortality rate if misdiagnosed or underestimated. Accurate differential diagnosis is of paramount importance (GoR moderate based on low LoE). - Treatment of cytomegalovirus colitis should be nonoperative, including antiviral therapy, broad-spectrum antibiotics, and bowel rest. Emergency surgery must be reserved only for those patients presenting with signs of toxic megacolon, fulminant colitis, perforation, or ischemia (GoR moderate based on low LoE).
***Clostridioides difficile*** **colitis**	- A damage control approach in complicated cytomegalovirus colitis should be adopted in severely sick patients with physiological derangement (GoR moderate based on low LoE). - No sufficient data exist to indicate whether to perform subtotal or segmental colectomy resecting only the involved colon segment. - Patients with severe *Clostridioides difficile* colitis who progress to systemic toxicity should undergo appropriate medical treatment and early surgical consultation (GoR moderate based on intermediate LoE). - Resection of the entire colon should be considered in the treatment of patients with fulminant colitis (GoR moderate based on intermediate LoE). - Diverting loop ileostomy with colonic antibiotic lavage is an effective alternative to subtotal colectomy (GoR moderate based on intermediate LoE). - A damage control approach in severe *Clostridioides difficile* should be adopted in severely sick patients with physiological derangement (GoR moderate based on low LoE).
**Common acute abdominal infections in transplanted patients**	
	In transplanted patients, the epidemiology of acute surgical diseases varies, with gallbladder disease being one of the most common problems after heart and/or lung transplantation and intestinal perforation due to diverticulitis being the most common disease following kidney and liver transplants (GoR moderate based on intermediate LoE).
**Acute cholecystitis**	- Laparoscopic cholecystectomy is feasible and should be preferred whenever possible in transplanted patients experiencing acute cholecystitis (GoR moderate based on intermediate LoE). - Transplanted patients with acute cholecystitis should undergo cholecystectomy as soon as possible after the diagnosis (GoR moderate based on intermediate LoE). - Percutaneous cholecystostomy may be a useful temporary or permanent procedure in patients with acute cholecystitis of both calculous and acalculous origin, who are unfit for surgery (GoR moderate based on intermediate LoE). - Prophylactic cholecystectomy in patients who are candidates for transplantation may be considered in selected patients (GoR weak based on low LoE)**.**
**Acute appendicitis**	- There is no data to recommend conservative treatment of acute appendicitis in transplanted patients. Given the high rate of complicated appendicitis and the good clinical outcomes observed after surgical intervention, operative management may be considered safer (GoR weak based on low LoE). - Transplanted patients with acute appendicitis should undergo appendectomy as soon as possible and usually within 24 h from the diagnosis (GoR moderate based on intermediate LoE). - Laparoscopic appendectomy should be preferred whenever feasible and not contraindicated (GoR moderate based on intermediate LoE).
**Acute diverticulitis**	- Acute left side colonic diverticulitis is associated with increased mortality in immunocompromised patients. Accurate diagnosis and follow-up are mandatory in this cohort of patients (GoR moderate based on intermediate LoE). - Kidney and liver transplanted patients, as well as patients on immunosuppressant drugs (chronic steroid/immunosuppressant therapy), have higher incidence and higher severity of acute colonic diverticulitis compared to the general population (GoR moderate based on intermediate LoE). - Transplanted patients admitted for acute uncomplicated colonic diverticulitis may receive a trial of medical therapy with bowel rest, intravenous antibiotics, and supportive care (GoR moderate based on intermediate LoE). - When complicated acute colonic diverticulitis occurs in transplanted patients, or the patients fail to improve with medical therapy, surgical intervention is indicated. It should be performed as soon as possible from the decision to operate (GoR moderate based on intermediate LoE). - Emergency surgery for acute left side colonic diverticulitis is associated with higher mortality and morbidity in immunocompromised patients (GoR moderate based on intermediate LoE). - Hartmann procedure is effective and safe in severely sick immunocompromised patients affected by acute left side colonic diverticulitis (GoR moderate based on intermediate LoE). - Damage control approach is a viable alternative in severely sick immunocompromised patients affected by acute left side colonic diverticulitis in which it is not feasible to achieve complete source control or whenever an abbreviated surgical procedure is required by clinical conditions (GoR moderate based on low LoE) - No sufficient data exist to define conditions for sigmoidectomy and primary anastomosis associated with a diverting ileostomy during emergency surgery for acute colonic diverticulitis in immunocompromised patients. - There are not sufficient data to support a laparoscopic over an open approach in acute complicated diverticulitis in transplanted patients. - Transplanted patients healed from an episode of uncomplicated acute diverticulitis do not require mandatory colic resection but should be advised about the slightly higher recurrence rate compared to the general population (GoR moderate based on intermediate LoE). - Elective sigmoidectomy may be proposed to immunocompromised patients after an episode of complicated acute left-sided colonic diverticulitis treated nonoperatively, especially after a recurrence (GoR moderate based on low LoE). - In transplanted patients, elective sigmoidectomy has a mortality and morbidity rate similar to the general population (GoR moderate based on intermediate LoE). - Patients with chronic kidney disease and/or patients on chronic steroid medication should be advised of the risk of having a more severe acute diverticulitis episode and may benefit from elective colectomy if fit for the procedure (GoR moderate based on intermediate LoE). - Adult polycystic kidney disease patients listed for kidney transplantation and with known diverticular disease should not be offered elective sigmoidectomy as a standard approach (GoR moderate based on intermediate LoE). If living donor transplantation is planned, the possibility of elective laparoscopic sigmoidectomy should be discussed with the patient.
**Patients with HIV/AIDS**	
	- HIV infection itself should not guide therapeutic decisions or prognostic counseling in patients with acute abdominal problems since most of the preoperative prognostic factors of HIV patients are similar to those of the general population (GoR moderate based on low LoE). - Patients with HIV should be stratified according to the current stage of the disease and the presence or absence of AIDS-defining conditions, as well as the associated prognostic factors (GoR moderate based on low LoE). - CD4 count and viral load should always be measured in HIV/AIDS patients undergoing emergency abdominal surgery in an attempt to predict a higher rate of postoperative complications (GoR moderate based on intermediate LoE). - HIV-infected patients with normal CD4 count (> 200 cells/mm^3^) have mortality and morbidity rate similar to the general population (GoR moderate based on intermediate LoE). - Worse perioperative outcomes have been observed in HIV/AIDS patients with lower CD4 count and higher viral load (GoR moderate based on intermediate LoE). - HIV and AIDS patients should continue antiretroviral therapy per os as long as possible when an indication for surgery exists. If suspended, they should resume it as soon as possible after surgical intervention (GoR moderate based on intermediate LoE).
**Perioperative steroid management**	
	- In patients currently on steroid therapy or that have been in steroid therapy for the last year, there is no evidence regarding the necessity of the administration of a push-dose steroid in the event of a surgical intervention (GoR moderate based on intermediate LoE). - No sufficient data exist to suggest the suspension of steroid medication before emergency surgery. Patients on steroids should remain on their usual regimen, and the treating physician should be aware of a higher rate of surgical complications when planning the intervention (GoR moderate based on low LoE). - In the event of an inexplicable and fluid unresponsive hypotensive event immediately prior/after/during surgery, adrenal insufficiency should be part of the differential diagnosis and an i.v. push dose of 100 mg hydrocortisone should be administered (GoR moderate based on low LoE).

### Definitions

#### Definition of the immunocompromised patient

An immunocompromised host is a patient presenting an impaired or weakened immune system; this does not allow a normal response to infections.

Immunocompromised patients are defined as follows [5]:

1. Congenital conditions (T- or B-cell defects, macrophage dysfunctions, often in newborns and children but even in the adult population)
2. Acquired conditions
  - Infected by human immunodeficiency virus (HIV) who developed acquired immunodeficiency syndrome (AIDS)
  - Hematologic malignancy
  - Patients affected by intrinsic immune conditions considered immunodeficiency along with one between “solid malignancy or solid organ transplanted patients or inflammatory disease/rheumatologic disease” plus the concurrent assumption of immunomodulatory drugs or chemotherapy
  - Patients in a physiologic or pathologic condition that is accompanied by any degree of immunodeficiency (Table 1)

#### Classification of immunodeficiency state

Table 1 shows the conditions causing immunodeficiency, ranging from mild to severe.

### Notes on the use of the guideline

The guidelines are evidence-based, with the grade of recommendation based on the evidence. The guideline presents the diagnostic and therapeutic methods for optimal management of acute abdomen in the immunocompromised patient. The practice indications promulgated in this work do not represent a standard of practice. These are suggested plans of care based on the best available evidence and experts’ consensus, but they do not exclude other approaches as being within the standard of practice. For example, they should not be used to compel adherence to a given medical management method, which method should be finally determined after taking account of the conditions at the relevant medical institution (staff levels, experience, equipment, etc.) and the characteristics of the individual patient. However, the treatment results’ responsibility rests with those directly engaged and not with the consensus group.

## Diagnosis

Diagnosis and treatment in immunocompromised patients must be multidisciplinary (GoR moderate based on low LoE).

High clinical suspicion must be kept in the presence of an immunocompromised patient presenting with signs and/or symptoms of possible intrabdominal infection (GoR moderate based on low LoE).

Immunocompromised patients usually do not present specific signs and symptoms. A reliable diagnosis may be reached only by combining signs, symptoms, patient history, and imaging evaluation (GoR moderate based on low LoE).

Clinical signs may not be reliable in immunocompromised patients; the more the immunocompromission, the less the reliability (GoR moderate based on low LoE).

Laboratory tests may not accurately reflect the severity of the clinical condition of the patient immunocompromised (GoR moderate based on low LoE).

Plain radiographs and ultrasound are often not sufficiently sensitive and specific to allow for a definitive diagnosis in immunocompromised patients (GoR moderate based on low LoE).

Contrast-enhanced CT scan, whenever feasible, is the most reliable exam to diagnose intrabdominal disease in immunocompromised patients (GoR moderate based on low LoE).

In the event of diarrhea, with or without acute abdomen, a specific test for *Clostridioides difficile* and its toxin should be performed (GoR moderate based on low LoE).

Additional microbiologic tests for a specific disease should be performed only if clinically congruent (GoR moderate based on low LoE).

Diagnostic workup for acute abdomen in patients with HIV infection should always consider surgical diseases specifically associated with HIV (i.e., Abdominal tuberculosis, Mycobacterium avium complex infections) (GoR moderate based on low LoE).

IC patients’ status at presentation may vary from reasonably functional and able to carry on daily activities, to extreme physical debilitation, with inadequate nutrition, considerable pain, and other significant comorbidities. Along with a thorough history and physical examination, further laboratory evaluations and tests include, but are not limited to, a complete blood count, serum electrolytes, liver function tests, and coagulation studies. C-reactive protein (CRP) may become fundamental in differential diagnosis. Depending on the degree of cardiac involvement and type of surgery planned, a 12-lead ECG and echocardiogram may be advisable. A chest radiograph should be considered to screen for tuberculosis, metastatic intrathoracic disease, pleural effusions, or other pulmonary disease processes that may have perioperative consequences.

Fever, leukocytosis, and peritonitis may be mild or absent, especially in patients with severe IC [6].

A first-level radiological evaluation with US and X-ray may not be sufficiently effective in obtaining a definitive diagnosis. Since IP mortality is higher if a diagnosis of surgical disease is missed, liberal use of contrast-enhanced CT scan is advocated for this population [7].

## Specific acute abdominal infections in immunocompromised patient

### Neutropenic enterocolitis

Statements are as follows:

- Neutropenic enteritis and typhlitis have a high mortality rate if misdiagnosed or underestimated; accurate differential diagnosis is mandatory (GoR moderate based on low LoE).
- Treatment of neutropenic enteritis and typhlitis should be nonoperative, including broad-spectrum antibiotics and bowel rest. Emergency surgery must be reserved only for those patients presenting with signs of perforation or ischemia (GoR moderate based on low LoE).
- A damage control approach in complicated neutropenic enteritis and typhlitis should be adopted in severely sick patients with physiological derangement (GoR moderate based on low LoE).

Neutropenic enterocolitis (ileocecal syndrome or typhlitis) is the commonest cause of acute abdominal pain in neutropenic cancer patients. Typically, it occurs 1 or 2 weeks after chemotherapy is initiated [8] and is more common in leukemic patients or patients after high-dose chemotherapy for solid organ cancer [9]. Almost 1% of all cancer patients admitted to the emergency department yearly had neutropenia at the admission [7] and 6.5% of neutropenic patients for myelosuppressive therapy have neutropenic enterocolitis. Four percent of cancer patients admitted to emergency departments had neutropenic fever [10]. Up to 7% of cancer-related ICU admissions are for neutropenic patients [11]. The real incidence of neutropenic enteritis ranges from 0.8 to 26% [7, 12].

Neutropenic enterocolitis generally presents with neutropenia associated with one or more of the following signs and symptoms: fever, bowel wall thickening, diarrhea, and abdominal pain [13–15].

US signs that increase the risk of complications are fluid-filled bowel, ascites, free fluid between bowel loops, and hyperechoic septa floating inside the bowel’s lumen (that correspond to bowel necrotic mucosa).

Half of the patients with signs and symptoms of neutropenic enteritis have an ultrasound positive for bowel wall thickening (> 5 mm), confirming the diagnosis. Up to 70% of patients with a positive US have a full recovery after a mean of 8 days; 100% of the patients without identified bowel wall thickening have a full recovery after an average of 4 days [15]. Patients with US scan positive for bowel thickening > 10 mm had a higher death rate [13]. Mortality in patients with US or CT scan positive for suggestive signs of neutropenic enteritis or typhlitis reaches 29.5%. Therefore, a high index of suspicion in patients undergoing conservative treatment with positive radiologic signs is mandatory (see diagnosis paragraph for high-risk radiological signs).

CT scan detection of right colon wall thickening is the best indicator of the diagnosis and a good predictor for the prognosis. Patients with bowel wall > 10 mm had a 60% risk of death compared to 4.2% if < 10 mm [16]. Once the diagnosis is confirmed, immediate broad-spectrum antibiotic therapy must be initiated. The disease should be treated with empiric antimicrobial therapy according to the IDSA guidelines for “fever with neutropenia” [17]. They suggest monotherapy with an anti-pseudomonas B-lactam agent or a carbapenem or piperacillin-tazobactam as the first choice. The addition of other antimicrobials may be suggested if no clinical improvement is observed and/or if a specific infection focus is suspected and/or in case of complications. No indications for the immediate administration of empirical antifungal therapy exist [18]. Adjunct antifungal therapy may be added if fever failed to improve after empiric antibiotic therapy. Resolution is obtained in up to 86% of patients with conservative antibiotic treatment in a median of 6–8 days. Interestingly, a rise in the neutrophil count after nadir would directly correlate with the resolution of symptoms [15, 18, 19].

Treatment of neutropenic enteritis or typhlitis is nonoperative with antibiotics and bowel rest [20]. Surgery must be reserved only for those presenting with signs of perforation or ischemia.

No studies investigated surgical vs*.* conservative management of patients with neutropenic enteritis, but it is widely accepted that conservative management should be preferred. Cancer patients developing neutropenic enteritis, usually after high-dose chemotherapy, are poor candidates for surgery, especially if unplanned. Neutropenic enteritis generally develops during the second-third week of chemotherapy (the period of the mucosal damage induced by drugs) [21].

After chemotherapy, in a spot of 30 days, it has been shown that planned elective surgery does not carry excessively higher risk [22]. Conversely, on chemotherapy, the reported mortality rate is up to 81%. Patients with leukemia who underwent emergency surgery and had chemotherapy in the previous 30 days presented a 57% mortality rate, with leukopenia being an adverse prognostic factor [23].

Comparing patients who had chemotherapy in the previous 30 days undergoing emergency surgery to those who had not, mortality and complication rates were higher in the chemotherapy group (22.4% vs. 10.3% and 44% vs. 39.2%, respectively). Leukopenia (WBC count < 4500 × 10^3^/mm^3^) was associated with a higher risk of mortality and morbidity (24.4% vs. 10.8% and 45.4% vs. 26.9%, respectively) [24].

Concerns may exist in admitting patients to the ICU with ongoing cancer progression or recurrence after emergency surgical intervention. Indication for ICU admission should be defined on a case-by-case basis, considering all the clinical, organizational, and even economic aspects. A large multicenter study on 717 cancer patients admitted to 28 different ICUs reported a rate of in-hospital mortality for emergency surgery of 37%. In contrast, ICU mortality for the same category was 23%. Mortality was related to the need for mechanical ventilation and performance status and not directly to cancer-related characteristics [11].

### Cytomegalovirus colitis

Statements are as follows:

- Cytomegalovirus colitis has a high mortality rate if misdiagnosed or underestimated. Accurate differential diagnosis is of paramount importance (GoR moderate based on low LoE).
- Treatment of cytomegalovirus colitis should be nonoperative, including antiviral therapy, broad-spectrum antibiotics, and bowel rest. Emergency surgery must be reserved only for those patients presenting with signs of toxic megacolon, fulminant colitis, perforation, or ischemia (GoR moderate based on low LoE).
- A damage control approach in complicated cytomegalovirus colitis should be adopted in severely sick patients with physiological derangement (GoR moderate based on low LoE).
- No sufficient data exist to indicate whether to perform subtotal or segmental colectomy resecting only the involved colon segment.

Cytomegalovirus (CMV) infection accounts for up to 34% of severe acute colitis in IC. Liver transplant recipients have been described to have a 4.9% 10-year cumulative incidence of post-transplantation CMV end-organ disease (colitis, hepatitis, pneumonia) [17]. After allogeneic hematopoietic stem cell transplantation, the incidence of CMV end-organ disease is 15–25% [18]. Even HIV-positive patients with or without AIDS, kidney transplant recipients, and patients with malignancies may present with severe CMV infections. In pediatric patients, the most common cause is acute lymphoblastic leukemia [25]. CMV colonic localization is the most common and causes vasculitis that ultimately leads to bowel wall necrosis. CMV colitis symptoms are nonspecific, encompassing all mild-to-severe colitis symptoms like diarrhea, rectal bleeding, fever, abdominal pain, weight loss, and up to colonic perforation [26, 27]. Patients with CMV colitis usually do not present classical CMV viremia symptoms (pharyngitis, lymphadenopathy, splenomegaly) [18]. In diagnosing CMV colitis, blood serology has no diagnostic value. The CMV seroprevalence analysis in adults showed at least 70% of seropositivity [28, 29]. At the endoscopy, the only factor that may suggest the diagnosis is the presence of ulcerations with a well-defined, punched-out appearance present in up to 80% of patients [30–32]. Some studies proposed a typical cecum ulcer involving the ileocecal valve as a specific finding in CMV colitis in patients with graft-versus-host disease [33]. A biopsy is always required when colonoscopy is performed in IP, specifically considering CMV infection. In hematoxylin-eosin-stained tissue sections, the “owl eye” appearance inclusions and are highly specific for CMV. The “gold standard” for diagnosing CMV colitis is the CMV-specific immunohistochemistry in tissue biopsies [34].

Contrast-enhanced CT scan is helpful for the diagnosis. Bowel thickening is almost always present, but pancolic appearance is rare and may help in differential diagnosis with CDc together with the presence of small bowel thickening (present in up to 40% of CMV infections and absent in CDc) [35]. In-hospital mortality of immunocompetent severely ill patients with CMV colitis is almost 70% despite treatment [36]. Results in immunocompromised patients are even worst. The possible association between inflammatory bowel disease (IBD) and CMV colitis should be kept into consideration. In fact, patients affected by IBD presenting even a CMV colitis may experience up to seven times higher in-hospital mortality [37].

There are insufficient publications with good quality to determine if treating CMV colitis with antiviral agents will improve patient outcomes regarding colectomy and mortality rate. However, untreated CMV disease in immunodeficient patients is associated with higher morbidity and mortality. The drug of choice for initial therapy in adults is intravenous ganciclovir (5 mg/kg twice daily) [38, 39]. After 3–5 days of intravenous ganciclovir, a transition can be made to oral valganciclovir (900 mg/twice daily) for the remainder of the 2–3-week course [40]. In pediatric patients, 14–21 days of parenteral ganciclovir is recommended. Early switch to oral treatment in children may promote CMV reactivation [41]. Large spectrum antibiotic therapy is indicated.

A subtotal or partial colectomy is indicated in severe conditions characterized by toxic megacolon, fulminant colitis, perforation, or ischemia. No definitive data exist in defining the superiority of segmental colectomy over subtotal colonic resection.

### *Clostridioides difficile* colitis

Statements are as follows:

- Patients with severe *Clostridioides difficile* colitis who progress to systemic toxicity should undergo appropriate medical treatment and early surgical consultation (GoR moderate based on intermediate LoE).
- Resection of the entire colon should be considered in the treatment of patients with fulminant colitis (GoR moderate based on intermediate LoE).
- Diverting loop ileostomy with colonic antibiotic lavage is an effective alternative to subtotal colectomy (GoR moderate based on intermediate LoE).
- A damage control approach in severe *Clostridioides difficile* should be adopted in severely sick patients with physiological derangement (GoR moderate based on low LoE).

*Clostridioides difficile* colitis (CDc) ranges from 6 to 33% in hematology-oncology population with most cases occurring in the first month post-transplantation [42–48]. In transplanted patient incidence ranged from 0.77 to 11.3% in kidney transplant (KT) up to 0.63 to 19% in liver transplant (LT) and 1.93 to 22.9% in lung transplant [49–55]. In HIV-infected patients, incidence is 7.1–8.3 cases 1000 patients/year [56, 57]. Common risk factors are generally the use of high-risk antibiotics such as antipseudomonal penicillin, fourth generation cephalosporins, carbapenems, fluoroquinolones, and clindamycin [43, 47]. Other risk factors included CD4 count ≤ 50 cells/μL [57] grade ≥ 2 mucositis [45, 47], higher dose of chemotherapy, reactivation of cytomegalovirus, and reactivation of other *Herpesviridae* [46]. Acute abdomen is rarely the first manifestation, but it may occur in combination with diarrhea, leukocytosis and fever. Radiological findings are various in CDc with normal X-ray of the abdomen in up to 68% [58].

Free fluid detected with ultrasound is present in CDc (77%) [59].

Contrast-enhanced CT scan has the best diagnostic power in detecting signs of CDc. It may be available before toxin stool testing and represents the gold standard if associated with signs and symptoms. Up to 84% of patients with CDc show at CT scan colonic wall thickening with 50% being pancolic [60, 61].

CDc infection is mainly a medical disease. Optimal timing for emergent surgical intervention remains controversial. Surgical management should be performed when the clinical conditions worsen or do not improve with maximal medical and supportive therapy. Patients with fulminant colitis progressing to systemic toxicity require emergent surgical intervention. The mortality rate of emergency surgery performed in patients with CDc is 35% [62] and higher survival rates are observed in patients managed in dedicated surgical units. Predictors of mortality include age > 70 years, severe leukocytosis or leukopenia (white blood cell count, ≥ 35,000/μL or < 4000/μL) or bandemia (neutrophil bands, ≥ 10%), cardiorespiratory failure, thrombocytopenia (platelet count < 150 × 100/mm^3^), coagulopathy (international normalized ratio > 2.0), and renal insufficiency (blood urea nitrogen > 40 mg/dL) [63, 64].

The effects of a short period of medical optimization before colectomy in improving outcomes are debated. At present, no clinical and/or laboratory findings exist able to predict neither who will improve with medical therapy nor who needs surgery [65]. The timing of surgical intervention is the most important factor influencing survival [66–69].

Subtotal colectomy is the intervention of choice and is superior to partial or segmental colectomy or other surgical procedures [62, 70]. Diverting loop ileostomy with antegrade colonic lavage with vancomycin may be a colon-preserving alternative to subtotal colectomy with good results regarding morbidity and mortality [71, 72].

*Intestinal tuberculosis (TB)* is one of the most common abdominal diseases in IP, especially in low resource settings [73–75]. Its diagnosis is generally difficult and may be based on local epidemiology. It may affect almost any intracavitary organ and has nonspecific symptoms in the majority of cases. Presentation symptoms and signs are generally aspecific: fever (75%), abdominal pain (65%), and weight loss (36%) had a higher prevalence than the other ones. The most frequent imaging findings are lymph-nodal disease (23%), gastrointestinal tract (19%), and solid organs (10%) involvement. In the gastrointestinal tract, the terminal ileum and the ileocecal region are the most affected (50%). Liver and spleen show greater involvement among solid organs (70%) [76].

Peritoneal tuberculosis is the most common form of abdominal tuberculosis and includes the peritoneal cavity, the mesentery, and the omentum. Free or loculated ascites can be present in 30–100% of cases and tomographic density is variable (20–45 UH), depending on the stage of the disease. Only 3% of patients have the dry type of tuberculosis peritonitis. Multiple mesenteric lymph nodes with peripheric enhancement and central hypodensity can be seen and aid in the diagnosis [3]. The presence of lipohydric level, in association with necrotic lymph nodes, is highly specific for tuberculous ascites [77]. Abdominal TB is generally characterized by three main presentations associated with several less specific symptoms: the ascitic, the plastic (which causes intestinal obstruction), and the glandular presentation (which involves the mesenteric nodules). Less commonly, it may be possible to observe tuberculous strictures, nodules, fistulae, or an interconnected association of these manifestations [75, 78]. Generally, CT scan is not sufficiently sensible or specific. Test for purified protein derivative is usually negative in IP. Additionally, up to 85% of patients with abdominal TB will not have any form of pulmonary involvement [79]. Differential diagnosis is fundamental in defining the presence of abdominal TB in IC. Treatment of intestinal TB is mainly medical. In case of complication as perforation, the treatment of choice seem to be resection and anastomosis more than direct suture of the perforation [78].

## Common acute abdominal infections in transplanted patients

In transplanted patients, the epidemiology of acute surgical diseases varies, with gallbladder disease being one of the most common problems after heart and/or lung transplantation and intestinal perforation due to diverticulitis being the most common disease following kidney and liver transplants (GoR moderate based on intermediate LoE).

Up to 30 % of transplanted patients frequently present to the ED with abdominal pain as the first complaint, but only 10% of them will require emergency surgery [80].

It is essential to consider the time from initiation of immunosuppressant therapy with the onset of abdominal pain. In fact, the longer the time from initiation of immunosuppressant therapy, the milder the signs and symptoms of the abdominal disease may be [81, 82].

Several common medical conditions may be responsible for infectious diseases in transplanted patients mimicking acute abdomen. The timeline from the transplantation and consequent initiation of immunosuppressive therapy may help in narrowing the differential diagnosi s[81].

*During the first month after transplantation,* suspicion should be highest for nosocomial infections related to the hospital stay and surgery. Incision cellulitis, intra-abdominal abscess, fungal infection, urinary tract infection, hospital-acquired/ventilator-associated pneumonia, *Clostridioides difficile*, or bacteremia secondary to central line placement should be ruled out [81].

*During months 1 to 6 after transplantation,* generally, the patient undergoes the greatest immunosuppression, and this timeframe is at the highest risk for opportunistic infections. Acute viral infections, such as CMV and bacterial infections similar to those discussed below for HIV-related acute abdominal conditions, may all be present in post-transplant patients during this time.

*After six months from the transplantation,* variability in the immune response is observed in this group of patients. For those requiring low-dose antirejection therapy, the risk of infection presenting as abdominal pain is similar to immunocompetent patients. Patients requiring a more intensive antirejection regimen continue to have a higher risk for opportunistic infections [81].

In general, abdominal pain and fever were the most common presentation. Conversely, leukocytosis was absent in 65% of these patients than 33% of immunocompetent ones [80, 83].

### Acute cholecystitis

Statements are as follows:

- Laparoscopic cholecystectomy is feasible and should be preferred whenever possible in transplanted patients experiencing acute cholecystitis (GoR moderate based on intermediate LoE).
- Transplanted patients with acute cholecystitis should undergo cholecystectomy as soon as possible after the diagnosis (GoR moderate based on intermediate LoE).
- Percutaneous cholecystostomy may be a useful temporary or permanent procedure in patients with acute cholecystitis of both calculous and acalculous origin, who are unfit for surgery (GoR moderate based on intermediate LoE).
- Prophylactic cholecystectomy in patients who are candidates for transplantation may be considered in selected patients (GoR weak based on low LoE).

Acute cholecystitis (AC) clinical signs such as pain in the right upper quadrant, temperature > 38 °C, and elevation in bilirubin levels have been reported up to 65%, 26%, and 10% of patients, respectively. Ultrasound signs of AC are present in up to 87% of patients. Acalculous AC accounts for up to 40% of cases, with a higher percentage concerning the general population. White blood cell count alteration occurred in almost 55% of patients, with C-reactive protein elevation in nearly 68% of cases [84].

AC frequently occurs after heart, lung, and kidney transplantation. The incidence is up to 72.2% after heart transplant and up to 30% after kidney transplant [85–89]. A large study evaluated 1687 *heart transplant* recipients undergoing cholecystectomy. 72.2% of patients had AC and were admitted urgently/emergently in the 60.9% of cases. Overall postoperative mortality was 2.2%. Open cholecystectomy was associated to higher morbidity and mortality compared to laparoscopic (6.2% vs. 0.9%; *P* = 0.009) as well urgent/emergent cases compared to elective cases (3.6% vs. 0%; *P* = 0.04) [85]. Acute post-transplantation urgent operation for acute complications of the biliary tract are associated to a mortality rate up to 29% [86].

Among 1595 *renal transplant* patients, 31 underwent laparoscopic cholecystectomy for AC with a conversion rate of 32.3%. Severe cholecystitis (empyema, phlegmon, or gangrene) was pathologically confirmed in 15 patients (48.4%). Acalculous AC was observed in 13 cases (41.9%). Overall morbidity was 19.4%. Surgical complications occurred in 12.9% of cases, with the need for reoperation in 2 patients (6.5%). There was no compromise of kidney function postoperatively. One graft was lost due to postoperative sequelae [84]. Prophylactic cholecystectomy before subsequent KT showed a mortality and morbidity rate of 0% and 12.5%, respectively [87].

Patients who undergo *allogeneic hematopoietic stem cell transplantation* (HSCT) are susceptible to infections, leading to increased morbidity and mortality. Acute cholecystitis is very common. Acute cholecystitis diagnosis is often delayed in the HSCT population because transplant patients are prone to multiple hepatobiliary complications with similar clinical presentations. The typical signs of infection may be masked by immune and marrow suppression. In the HSCT population, cholecystitis development was associated with an increased 1-year overall mortality rate (62.5% versus 19.8%, *P* < .001). Twenty cases of acute cholecystitis (62.5%) were treated with cholecystectom y[90, 91].

### Acute appendicitis

Statements are as follows:

- There is no data to recommend conservative treatment of acute appendicitis in transplanted patients. Given the high rate of complicated appendicitis and the good clinical outcomes observed after surgical intervention, operative management may be considered safer (GoR weak based on low LoE).
- Transplanted patients with acute appendicitis should undergo appendectomy as soon as possible and usually within 24 h from the diagnosis (GoR moderate based on intermediate LoE).
- Laparoscopic appendectomy should be preferred whenever feasible and not contraindicated (GoR moderate based on intermediate LoE).

The majority of patients had clinical symptoms and a suggestive CT scan, but only 25% of them showed leukocytosis. 8.2% of patients had complicated AA with perforation. IC patients with AA may show symptoms similar to the immunocompetent population, such as nausea/vomiting and fever along with right lower quadrant (RLQ) pain, but different laboratory pattern. Forty-three percent up to 76% of transplanted patients with AA had leukocytosis [92, 93], fever, or migrating pain, but all patients had elevated CRP [94]. Sarici et al. [95] conducted a case-control matched analysis confirming the incongruence in laboratory findings among transplanted patients with AA compared with non-immunocompromised patients. They found that LT patients with AA had median WBC count of 7.500 cells/mm^3^ vs. 12.500 in non-transplanted patients (*p* = 0.002) while CRP was 6.1 mg/dl vs. 0.8 (*p* = 0.009).

In *liver transplanted* patients, cumulative incidence of AA ranges from 0.09 to 0.54% [82, 92–94, 96] demonstrating the rarity of this pathology in LT. In a recent meta-analysis, AA accounted only for 2% of all emergency surgery in transplanted patients [97]. Jamtani et al. showed as early surgical intervention is mandatory in this population. No differences in outcome exist comparing laparoscopic to the open approach suggesting that laparoscopic appendectomy is feasible. Some series of post-transplantation AA showed a very low rate of perforated appendicitis at the specimen in those patients operated within 24 h from the insurgence of the symptoms. On the other hand, all the patients with perforated AA were operated after a median time of 72 h. Patients who underwent surgical procedures showed a rate of complication ranging around 25% [93].

In *kidney transplanted* patients, the incidence of AA is low [98]. Leukocytosis is rare in KT patients developing AA, but CRP result generally elevated [99]. Fifty percent of KT patients operated for AA had perforated AA and then resulted in a longer hospital stay. Those who had complicated AA experienced generally a longer time from diagnosis to surgical intervention than patients who had acute non-complicated appendicitis (overall time to surgery 69 h vs. 25 h *p* < 0.05).

### Acute diverticulitis

Statements are as follows:

- Acute left side colonic diverticulitis is associated with increased mortality in immunocompromised patients. Accurate diagnosis and follow-up are mandatory in this cohort of patients (GoR moderate based on intermediate LoE).
- Kidney and liver transplanted patients, as well as patients on immunosuppressant drugs (chronic steroid/immunosuppressant therapy), have higher incidence and higher severity of acute colonic diverticulitis compared to the general population (GoR moderate based on intermediate LoE).
- Transplanted patients admitted for acute uncomplicated colonic diverticulitis may receive a trial of medical therapy with bowel rest, intravenous antibiotics, and supportive care (GoR moderate based on intermediate LoE).
- When complicated acute colonic diverticulitis occurs in transplanted patients, or the patients fail to improve with medical therapy, surgical intervention is indicated. It should be performed as soon as possible from the decision to operate (GoR moderate based on intermediate LoE).
- Emergency surgery for acute left side colonic diverticulitis is associated with higher mortality and morbidity in immunocompromised patients (GoR moderate based on intermediate LoE).
- Hartmann procedure is effective and safe in severely sick immunocompromised patients affected by acute left side colonic diverticulitis (GoR moderate based on intermediate LoE).
- Damage control approach is a viable alternative in severely sick immunocompromised patients affected by acute left side colonic diverticulitis in which it is not feasible to achieve complete source control or whenever an abbreviated surgical procedure is required by clinical conditions (GoR moderate based on low LoE)
- No sufficient data exist to define conditions for sigmoidectomy and primary anastomosis associated with a diverting ileostomy during emergency surgery for acute colonic diverticulitis in immunocompromised patients.
- There are no sufficient data to support a laparoscopic over an open approach in acute complicated diverticulitis in transplanted patients.
- Transplanted patients healed from an episode of uncomplicated acute diverticulitis do not require mandatory colic resection but should be advised about the slightly higher recurrence rate compared to the general population (GoR moderate based on intermediate LoE).
- Elective sigmoidectomy may be proposed to immunocompromised patients after an episode of complicated acute left-sided colonic diverticulitis treated nonoperatively, especially after a recurrence (GoR moderate based on low LoE).
- In transplanted patients, elective sigmoidectomy has a mortality and morbidity rate similar to the general population (GoR moderate based on intermediate LoE).
- Patients with chronic kidney disease and/or patients on chronic steroid medication should be advised of the risk of having a more severe acute diverticulitis episode and may benefit from elective colectomy if fit for the procedure (GoR moderate based on intermediate LoE).
- Adult polycystic kidney disease patients listed for kidney transplantation and with known diverticular disease should not be offered elective sigmoidectomy as a standard approach (GoR moderate based on intermediate LoE). If living donor transplantation is planned, the possibility of elective laparoscopic sigmoidectomy should be discussed with the patient.

The incidence of acute colonic diverticulitis (AD) in transplanted ranges around 1–2% [100]. It is wrongly thought to be even higher in patients with adult polycystic kidney disease (ADPKD), where colonic diverticula are often present and could be in a non-conventional location (e.g., 39% of colonic diverticula in ADPKD were located in the right colon) [101–103]. Transplant patients have a 22-fold higher risk of experience complicate AD compared to the overall population [104] and generally develop it at a younger age (54 vs. 61 years *p* = 0.02) [105]. Patients with a known diverticular disease before transplantation have a 16% risk of developing AD after transplantation and the start of immunosuppression [105]. Usually, diverticulitis episodes in transplanted patients occur early after transplantation, the most of them within 2 years [106].

The rate of surgical intervention for AD in the general population ranges from 14 to 39% [107], in transplanted patients, is up to 94% of patients admitted for AD [105]. Reported overall morbidity and mortality rates after emergency surgery for AD in the general population are up to 24% and 5.7%, while in transplanted patients are up to 51% and 23% respectively [108].

Among IC admitted for left colonic AD and divided according to the cause of immunosuppression (steroids, transplant, cancer, etc.), the highest rate of immediate emergency surgery was observed in patients on chronic steroid therapy [66].

In general, patients rarely present with generalized peritoneal irritation signs; patients with free peritoneal perforation can manifest little or no abdominal symptoms and less severe leukocytosis [108, 109]. Up to 61% of TP needing admission for AD present complicated disease (45% for perforation and 16% colo-vesical or colo-vaginal fistula). General population with complicated AD varies from 14 to 19% [107].

In transplanted patients, elective sigmoidectomy may be considered after the first episode of AD, given the high morbidity and mortality rate when emergency surgery is required [108]. Biondo et al., in a series of 931 patients with AD, found that 22.9% of them underwent emergency surgery ad first admission [110]. IC patients had a more severe presentation (48.2% vs. 37.3% *p* < 0.009) compared with non-IC and resulted in a higher rate of upfront surgical treatment (31% vs. 21% *p* = 0.004). Of the 239 patients who underwent emergency surgery, 48 died, 33% in the IC group vs 15.9% (*P* = 0.004); fatality rate for patient treated conservatively was 3.5% vs. 1% (*p* = 0.03).

Klarenbeek et al. evaluated 291 patients to differentiate those who may benefit from elective surgery after nonoperative management of an AD episode [111]. Eighty-eight patients (30.2%) experienced recurrence after the first episode of AD. The mortality rate was 13%, with patients with perforation accounting for 80% of the deaths. Perforation was more common among those on immunosuppressant therapy (95% were on steroids), chronic renal failure, and collagen vascular disease. For this reason, elective sigmoidectomy in this population should be considered.

The need for elective sigmoidectomy after a successfully treated episode of acute uncomplicated diverticulitis in IC patients ranges between 20.7 and 30.2% of patients [110, 111] AD recurrence rate varies from in 21.5 to 27.8% in IC patients and 13 to 20.5% of non-IC. When recurrence occurred, it was more severe in the IC group (46% of IC vs. 15% in non-IC group) [66]. However, most cases (66.7%) were mild, and 7.1% of recurrence needed emergency surgery, similarly to the general population [112]. Elective sigmoidectomy (IC and non-IC) showed a mortality and morbidity of 0% and 17% respectively with no differences between the two groups.

Among kidney transplant (KT) patients, Catena et al. analyzed 1611 patients in 31 years and found 47 gastrointestinal perforations (prevalence 2.9%); 21 were colonic, and 90% of these occurred in ADPKD patients. In general, half of all perforations happened within the first year after KT, when immunosuppressant drug doses are higher [113]. The association of immunosuppressants and corticosteroids increases the risk of developing complicated AD. Hospital mortality for KT patients who experience AD can be very high, ranging from 19 to 100% [101, 103, 105, 108]. Mortality is influenced by the timing of intervention with patients operated on < 24 h from symptoms beginning showing better outcomes [104, 114]. A series of 1137 kidney transplant patients reported complicated AD in 46% of ADPKD patients with a rate of emergency surgical intervention of 52.9% [109]. The rate of complicated AD was higher in ADPKD compared with non-PKD patients (5.6% vs. 0.68%) [102]. Opposite results were also published [115]. Elective sigmoidectomy in patients with ADPKD before kidney transplant should consider the incidence of AD, which ranges between 0.9 and 1.25% [102, 105, 109, 115, 116]. In some studies, the mortality rate of kidney transplant patients operated on for AD is 0% [108, 109] therefore, the “on-demand” strategy seems to be safer. Lastly, patients who are candidates for living donor kidney transplants have never been assessed for this topic.

The choice between Hartmann’s procedure (HP) and resection and primary anastomosis (RPA) with or without protective loop ileostomy is debated even in non-IC patients. However, some recent evidence favors RPA over HP in hemodynamically stable patients [117, 118].

Dalla Valle et al. reported resection and primary anastomosis for two KT patients with AD experienced uneventful recovery; they had a 12.5% rate of mortality in a patient who underwent Hartmann procedure (HP) [115]. Scotti et al. [109], on the other hand, had 0% mortality, and every patient that needed surgical intervention had a Hartmann procedure.

Biondo et al. reported that IC patients operated on during the first AD episode underwent an anastomosis less frequently than those considered immunocompetent (27 vs. 64% *p* < 0.001). This could be explained by a higher number of Hinchey III/IV patients in the IC group (65 % vs. 40% p ≤ .001).

### Other transplant-related diseases

*Small bowel lymphoma* may occur in up to 46% of patients with AIDS or transplanted patients on a high dose of immunosuppressant drugs and may cause gastrointestinal perforation or bleeding. Moreover, *Kaposi sarcoma (KS)* or intestinal lymphoma may lead to intussusception, abdominal obstruction, and acute abdomen. When KS presents as intra-abdominal disease, usually there are also skin manifestations. Bright contrast enhancement of lymph nodes at CT can help diagnose KS; the presence of this sign has a positive predictive value of 79% [3]. Up to 50% of intestinal perforation in patients with a kidney transplant occurs in the first three months from the transplant.

*Graft-versus-host disease (GVHD)* more frequently develops in patients after allogeneic bone marrow transplantation; up to 20% of patients with GVHD will develop a gastrointestinal emergency such as perforation or hemorrhage [119]. Abdominal pain as the presenting symptom is atypical. The skin and gastrointestinal tract are the most commonly affected areas. Generally, within 2 to 6 weeks after the transplant, skin rash with diarrhea or less frequently associated with abdominal pain should raise suspicions [120, 121].

## Patients with HIV/AIDS

Statements are as follows:

- HIV infection itself should not guide therapeutic decisions or prognostic counseling in patients with acute abdominal problems since most of the preoperative prognostic factors of HIV patients are similar to those of the general population (GoR moderate based on low LoE).
- Patients with HIV should be stratified according to the current stage of the disease and the presence or absence of AIDS-defining conditions, as well as the associated prognostic factors (GoR moderate based on low LoE).
- CD4 count and viral load should always be measured in HIV/AIDS patients undergoing emergency abdominal surgery in an attempt to predict a higher rate of postoperative complications (GoR moderate based on intermediate LoE).
- HIV-infected patients with normal CD4 count (> 200 cells/mm^3^) have mortality and morbidity rate similar to the general population (GoR moderate based on intermediate LoE).
- Worse perioperative outcomes have been observed in HIV/AIDS patients with lower CD4 count and higher viral load (GoR moderate based on intermediate LoE).
- HIV and AIDS patients should continue antiretroviral therapy per os as long as possible when an indication for surgery exist. If suspended, they should resume it as soon as possible after surgical intervention (GoR moderate based on intermediate LoE).

In the last decade, the medical literature has been unclear if the high mortality observed in patients with HIV infection or AIDS derives from the inability of this susceptible population to tolerate emergent surgical interventions or whether the natural course of the disease leads to higher mortality rates. For this reason, knowing the HIV status of emergency surgery patients is essential, and testing should be rapidly available [122]. The initial data on mortality and morbidity of HIV/AIDS patients undergoing surgical intervention was obtained mixing HIV-infected and AIDS patients, leading to a misperception and wrong approaches to surgical intervention in HIV patients. More recent evidence shows that HIV patients with early infection or in early stages (e.g., CD4 > 500 and absence of AIDS-defining infections) have the same operative risk as HIV-negative patients and should therefore be treated accordingly [123–125].

Patients affected by AIDS admitted to ED for acute abdominal pain are a diagnostic challenge with a large spectrum of possible diagnoses [126, 127]. Surgeons must discriminate between HIV-infected patients with an unrelated surgical disease and abdominal conditions primarily related to HIV/AIDS. Abdominal tuberculosis is frequently seen as a co-infection [128, 129].

In general, the presence of an AIDS-related disease requiring surgical exploration increases morbidity, and the mortality risk for emergency surgery rises from 15 to 45% [130].

Owotade et al. [131] showed that up to 25% of HIV patients would require some form of surgical intervention, either elective or emergent. It could be even more significant in a country where HIV is endemic: in a single center in Durban, South Africa, the seropositivity rate for HIV on 350 patients admitted to the surgical ward was 39% [132].

Anyway, when surgery or invasive procedures are needed, one must consider the elevated rate of postoperative complications that are more frequent in patients with low CD4 count or high viral load [123, 124, 133].

Deneve et al. [134], in a study on 77 patients with HIV/AIDS, found that 55% had at least one postoperative complication. There was a 30% mortality rate; patients with lower CD4 count (< 200 cells/mm^3^) had a higher risk for emergency surgical intervention and experienced higher morbidity and mortality rates.

Grubert et al. [135] compared a cohort of HIV-infected women with their un-infected matched respective undergoing an abdominal surgical procedure to assess postoperative complication rates. They found that HIV-infected cases are more prone to experience infectious postoperative complications (fever > 48 h requiring antibiotic treatment (12% vs. 1.7%, OR 8.1 *p* < 0.001)).

Morrison et al. [136] compared two cohorts, one of more than 1300 patients with HIV and another of HIV-negative patients, both admitted for trauma. The death rate was higher in HIV patients than HIV-negative but without reaching significance (5.6% vs. 4.6% *p* = 0.84). After stratifying for age and ISS, it appears that HIV status did not affect mortality in any subclass except for patients older than 65 years (mortality in HIV+ 15.6% versus 8.5% in HIV− *p* = 0.001). Unfortunately, this work did not consider HIV infection severity, AIDS status or CD4 count, and viral load.

In patients undergoing antiretroviral therapy (ART), the mortality and morbidity rate after surgical intervention is lower than observed in HIV+ patients not undergoing ART. It is mainly influenced by the efficacy of the therapy that affects CD4 count. King Jr. et al. demonstrated comparable morbidity and mortality rate after surgical intervention in patients undergoing ART and with a CD4 count > 200 cells/mm^3^ [137].

Sandler et al. analyzed a large series of patients undergoing emergency surgery [138]. Their propensity score analysis compared HIV-positive patients without AIDS, AIDS patients, and HIV-negative patients. HIV-positive patients without AIDS had the same outcomes as HIV-negative patients. AIDS is the only factor influencing the prognosis. Mortality rates were 4.4%, 0.5%, and 1.6% for AIDS patients, HIV-infected patients, and HIV-negative patients. HIV-positive patients without AIDS showed lower mortality because they are usually younger without other comorbidities. HIV-negative patients undergoing emergency surgery in this cohort were older, with more comorbidities. The PRO-HIV study confirmed these results [139]. They reported an overall “adverse surgical outcome” identified by death or major infective complication of 6.6% in HIV-positive patients (mostly on ART therapy at the time of surgery). Urinary tract infections, pneumonia, and surgical site infections were the most frequent infective complications. Thirteen percent of blood cultures resulted positive in patients with postoperative fever.

Highly active antiretroviral therapy (HAART), along with current therapeutic options, improved the outcome of HIV patients after surgery. However, ART administration is per os [140] and patients undergoing emergency surgery are often in a nihil per os state, and pathology affecting the GI tract can impair intestinal absorption of ART drugs. For these reasons, it was argued if the absence of ART administration could increase the viral load, decrease CD4 count, and increase postoperative complications in HIV patients. Intravenous Albuvirtide may be an alternative in patients candidates for emergency surgery in which ART therapy cannot be initiated postoperatively [141].

## Perioperative steroid management

Statements are as follows:

- In patients currently on steroid therapy or that have been in steroid therapy for the last year, there is no evidence regarding the necessity of the administration of a push-dose steroid in the event of a surgical intervention (GoR moderate based on intermediate LoE).
- No sufficient data exist to suggest the suspension of steroid medication before emergency surgery. Patients on steroids should remain on their usual regimen, and the treating physician should be aware of a higher rate of surgical complications when planning the intervention (GoR moderate based on low LoE).
- In the event of an inexplicable and fluid unresponsive hypotensive event immediately prior/after/during surgery, adrenal insufficiency should be part of the differential diagnosis and an i.v. push dose of 100 mg hydrocortisone should be administered (GoR moderate based on low LoE).

Chronic steroid therapy (CST) is considered 20 mg/day prednisone or equivalent for at least 3 weeks [142, 143]. High corticosteroid doses have been routinely administered perioperatively as “push dose” (or stress dose) to patients on long-term steroid therapy. No evidence exist supporting this practice [144]. Recent reports concluded that “push-dose steroids” are not needed as long as the patient on high-dose chronic steroid therapy continues to assume their usual dosage [145–149].

Perioperative stress steroid dose, however, is frequently used by anesthesiologists to reduce and prevent such dramatic effects in the postoperative period [150–152]. The most followed practical recommendation is to administer 200 to 300 mg of hydrocortisone during surgery. Evidence supporting this practice is insufficient [151, 153–156].

Friedman et al. [149] demonstrated the capability to increase endogenous steroid production in response to surgical stress patients on high doses of chronic steroids before orthopedic procedures.

The recent approach is not to administer a push dose of steroid perioperatively in patients with a low probability of hypothalamic–pituitary–adrenal axis (HPA) suppression. In case of hypotension related to the adrenal crisis in the perioperative period (or during surgery), a push dose of 100 mg hydrocortisone is administered, followed by a continued supplement of 50 mg hydrocortisone q6h [151]. At present, in some centers, for patients with documented or presumed (from high dosage chronic therapy) HPA suppression, perioperative stress-dose steroid administration is still utilized even in the absence of high-quality evidence since it appears to carry minimal risk compared to the risk of adrenal crisis [157]. It has to be noted that although testing of the HPA can reveal an adrenal insufficiency, it does not directly predict the possibility of perioperative hypotension or clinical manifestation and therefore should not guide treatment [145, 149, 155].

Zaghiyan et al. [158] randomly assigned patients on chronic steroids or treated with steroids during the previous year who were going to major surgery. No differences in postural hypotension or adrenal insufficiency were seen between those receiving high-dose glucocorticoids (hydrocortisone 100 mg intravenously three times daily) and low-dose glucocorticoids (the equivalent of their preoperative dose given intravenously) [158].

Steroid therapy is a well-known cause of augmented morbidity and mortality among surgical patients. In some cases, complications could be severe, such as an anastomotic leak or dehiscence [159, 160]. The rate of anastomotic leak in patients on chronic steroid therapy is up to 6.2%, versus 3.3% observed in elective colonic surgery [161]. In patients with ulcerative colitis undergoing complex reconstructive procedures, the use of diverting ileostomy in patients taking a preoperatively high dose of steroids is broadly accepted [162–164].

Chouairi et al. [142] in a multicenter retrospective analysis with more than 180,000 patients on CST compared — with a propensity score-matched analysis — outcomes of surgical patients with and without CST. The CST population showed a longer hospital stay and a higher complication, reintervention, readmission, and mortality rate.

Ritter et al. [165] analyzed 686 patients affected by ulcerative colitis undergoing complex reconstructive procedures. 4.2% had an anastomotic leak. In the “leak” group, 34% of patients had oral steroid taper after surgery vs. 14% in the “non-leak” group (*p* = 0.003). No effect on complication was noted when analyzing preoperative steroid therapy or IV taper immediately after surgery.

Slieker et al. in a prospective cohort study of 259 patients on steroids undergoing left-sided colorectal anastomosis, had a 7-fold increase in the risk of developing an anastomotic leak, with a 15% mortality if steroid therapy is ongoing, independently from the presence of a diverting stoma [166].

Intraoperative hypotension that cannot be adequately managed by conservative means (*e.g.,* decreasing depth of anesthesia, fluid resuscitation, vasopressor administration, and managing metabolic abnormalities) should raise suspicion for adrenal crisis, and a rescue dose of 100 mg of hydrocortisone IV should be administered, followed by continued supplementation of 50 mg of hydrocortisone IV every 6 h [167].

Often, there is no time to consider preoperative testing to determine HPAA integrity. Clinical judgment is required whether to administer stress-dose steroids based on the patient’s perioperative condition (*e.g.*, degree of hemodynamic stability) and surgical risk. It is reasonable, for example, to withhold glucocorticoids if the patient is otherwise healthy and stable preoperatively without signs or symptoms of Cushing disease, with a low threshold for administration of a rescue dose of steroids in the event of unexplained intra- or postoperative hypotension [157].

Hydrocortisone is the drug of choice for stress and rescue dose steroid coverage [168]. Growing body of data suggests administration of dexamethasone instead, having no mineralocorticoid activity and probably the same protective effect in short course.

### Perioperative and anesthesiologic management

Immunocompromised patients should be considered “frail”. They are exposed to an increased risk of complications [169]. Perioperative care of IC patients requires a deep understanding of immune system function and pharmacological implications. Multidisciplinary management is crucial [170]. No definitive data exist about anesthetic drugs’ effect on the immune system. However, anesthesiologists and ICU physicians must be aware of the immunosuppressive effects of the different drugs and procedures as listed in Table 3 [171].

**Table 3:** Side effects of immunosuppressive treatments in the perioperative management (OKT3, monoclonal antibodies directed against CD-3 antigen on the surface of human T-lymphocytes)

	Cyclosporine	Tacrolimus	Azathioprine	Steroids	Mycophenolate mofetil	Anti-thymocyte globulin	OKT3
Anemia			+		+		
Leucopenia			++		+	+	+
Thrombocytopenia					+		
Hypertension	++	+		+			
Diabetes	+	++		++			
Neurotoxicity	+	+		+			
Renal failure	+	++					
Anaphylaxis						+	+
Fever						+	+

In HIV-positive patients under general anesthesia, pharmacokinetic interactions of antiretroviral therapy with cytochrome 450 enzyme should be considered [172]. Drugs like etomidate, atracurium, remifentanil, and desflurane can be safely used as their metabolism is independent of the cytochrome 450 enzyme [172]. In the group of non-depolarizing drugs, it is preferable to use agents independent of kidney and liver function (cisatracurium, atracurium) or with a reversal medication (sugammadex).

Patients receiving cyclosporine as immunosuppressive therapy may require a smaller dose of non-depolarizing muscle relaxant, and the recovery time may be prolonged [173]. Strict precautions on infection prevention should be applied [174]. Whenever possible, Cytomegalovirus status should be checked. Even in the case of emergent procedures, a complete preoperative assessment including cardiopulmonary status, glomerular filtration rate, liver function, blood gas analyses, bleeding risk assessment, and electrocardiography monitoring throughout the whole surgical procedure is strongly suggested. Patients should be promptly covered and actively warmed upon arrival in the operating room; even mild hypothermia has been shown to disrupt clotting and increase postoperative infection rates [175]. Specific attention must be posed on patients with primary immunodeficiency syndromes as Ig infusion must be considered [176, 177]. The dose of immunosuppressive drugs in transplanted patients should be continued postoperatively. Daily monitoring of the steady-state blood level is recommended.

## Conclusions

The management of immunocompromised patients with acute abdomen must be multidisciplinary. Appropriate recognition and stratification of this particular cohort of patients with its proper risks and clinical peculiarities allow setting the correct diagnostic and therapeutic pathways as management should be individualized.

## Data Availability

Not applicable.

## Abbreviations

WSES: World Society of Emergency Surgery
IP: Immunocompromised patients
ED: Emergency department
IC: Immunocompromission
CRP: C-reactive protein
HIV: Human immunodeficiency virus
AIDS: Acquired immunodeficiency syndrome
CDc: *Clostridium difficile* colitis
CMV: Cytomegalovirus
AA: Acute appendicitis
AD: Acute diverticulitis
AC: Acute cholecystitis
KS: Kaposi sarcoma
GVHD: Graft-versus-host disease
KT: Kidney transplant
HT: Heart transplant
LiT: Liver transplanted
ADPKD: Adult polycystic kidney disease
HP: Hartmann’s procedure
RPA: Resection and primary anastomosis
ICU: Intensive care unit
CST: Chronic steroid therapy
HPA: Hypothalamic–pituitary–adrenal axis
ACTH: Adrenocorticotropic hormone
ART: Antiretroviral therapy
HAART: Highly active antiretroviral therapy
ALB: Albuvirtide
